# Ringworm

**Published:** 1892-03

**Authors:** M. B. Hutchins

**Affiliations:** Lecturer on Dermatology Atlanta Medical College, Atlanta, Ga.


### "RINGWORM." *

BY M. B. HUTCHINS, M. D.,
Lecturer on Dermatology Atlanta Medical College,
Atlanta, Ga.

The subject chosen may seem trivial, but it is really one of considerable importance. There are practically four varieties of ringworm met in practice, viz., the ordinary *ringworm of the skin*, as seen, almost exclusively, in children ; the variety which Hebra called "*eczema marginatum*," affecting the scroto-femora region or the axilla ; *ringworm of the scalp*, also rare, save in

*Read before Atlanta Society of Medicine, January 19, 1891.

children, and *ringworm of the beard*—or true "*barber's itch.*" They have all been proven to be due to the vegetable fungus known as the *tricophyton*. This fungus can be demonstrated in the scales from a patch of ordinary ringworm by placing them on the microscope slide mounted in ordinary liquor potassæ, and only a medium high power of microscope is necessary to its demonstration. As affecting the hairy parts the microscope shows the fungus both in the scales and hairs, when they are mounted in liquor potassæ—used because of its faculty of disintegrating the hairs and scales, and dissolving or saponifying any oil globules which may be present. Staining is not necessary. The fungus is composed of rounded spores like portions of the roe of fish, and of branching threads. The threads (mycelium) are the first growth, and the spores are formed at their end, the fruit, as it were. Where the fungus is growing rapidly the mycelial threads are said to be the more abundant, and where there is less activity of growth the spores are said to predominate. There are several fungous growths, some producing disease, others harmless, which are nearly identical with the tricophyton, and I believe, as do some others, that we could with difficulty diagnose a case of ringworm by the microscopic appearances alone, without seeing the case or without cultivating the fungus and observing the peculiarities of its growth.

In the scales from the lesions of ringworm we can see the fungous elements, either between or penetrating the scales themselves. In a case of ringworm of the beard or scalp we may not only see the fungus in the scales, and the rootsheath, but also penetrating the hairs themselves. I did not mention *ringworm of the nails*, it being very rare and probably impossible of diagnosis without a history of ringworm elsewhere and the aid of the microscope.

In order to carry out my intention of making this paper wholly practical, I will give the diagnostic points in each variety of the disease, as taken from my notes of cases seen in practice. Some of the cases have already been described in THE ATLANTA MEDICAL AND SURGICAL JOURNAL, hence will be detailed here as briefly as possible.

RINGWORM OF THE SKIN, OR TRICOPHYTOSIS CORPORIS.

CASE I.—Little girl of 7. Five days before consultation a small, reddish, rough scaly point appeared beneath the right eye. On the following day there were several lesions on the face and nose. When seen, there were lesions upon the forehead, face and neck ; a small one in each axilla and one on the left wrist. Most recent lesions averaged the size of a silver half dime, slightly red and scaly, or covered with thin sero-crusts. Increasing in size, the center cleared to normal in color and only slight scaling, while the border was raised, clearly defined and formed of vesicles or small crusts. These lesions were somewhat larger than a dime.

Two days after beginning treatment the lesions had declined to such an extent as to have lost their typical appearances, but there were one or two new ones. Brother and sister, aged respectively 4 and 9, had the same disease, proving its contagious character. They were ordered to use the treatment prescribed for the first. The original patient was practically well within a week of beginning of treatment. I heard later that some of the children had "relapses" now and then, but all finally recovered.

A case which was not at all typical was that of a lady, aged 29. This case has been reported, hence will be only briefly mentioned here. Disease began on left wrist, over a year before consultation, as "a little pimple," which slowly spread peripherally, clearing in the center, the border about one-twelfth of an inch width and of purplish color. No scaling, probably because of frequent washing. Always itchy when irritated. When examined there was a roundish dime-sized "spot" formed by the purplish border and clear, nearly normal center, while there was, touching this with borders broken at point of contact, a similar lesion the size of a split pea. The diagnosis was made in this case by the appearance alone, as the microscopic examination of epidermis revealed nothing. Treatment for ringworm was given and the patient finally recovered.

Another unusual case was that of the wife of one of our physicians. I must state that the doctor did not agree with me as to the diagnosis. I did not make notes of the case but will try and describe it from memory. On the back of the wrist was a sharply circumscribed patch about the size of a half dollar. This was marked by redness, and the formation of numerous scales or crusts. The patch had been present for some time and had resisted the treatment for eczema. It disappeared after I had used my usual treatment for ringworm, which the doctor followed with applications as for eczema. Crocker, the English dermatologist, says the patchy form of ringworm is about as common in adults as the ring form.

### RINGWORM OF THE SCROTO-FEMORAL REGION.

I have seen a number of these cases, and they are much more common than usually supposed. The following extensive case is a fair type. Teamster, aged 38. Disease began about anal fold two years before consultation. When examined, there were in the scroto-femoral region the usual stains left by the passage of the disease outward and downward. The staining terminated on left thigh at a large patch of the disease. Pubic region, and upward and nearly to the umbilicus also involved. Large patches posterior of both thighs, and the buttocks were entirely covered with the disease. The borders of the eruption were well defined, raised and composed of papulo-vesicles or minute crusts. Internal to the borders were a few more or less distinct and smaller rings, and numerous scratch marks. Redness and scaling on penis and scrotum. Whole diseased surface very itchy when patient was too warm, or upon exposure to the air.

This form of the disease usually begins in the fold between the scrotum and the thigh, thence spreading, with pigmented track behind, over adjacent surface, especially towards thighs and buttocks. It is rare for new patches to develop in the areas over which the disease has passed. The well-defined more or less rounded semi-circular border composed of vesiculo-crusted lesions is typical. Further away from the point of origin we may see the typical rings of ordinary cutaneous ringworm.

A rather surprising case was one which was recently treated. When first examined the sides of the scrotum, the scroto-femoral region and perineum were the seat of what appeared to be a chronic erythematous eczema, with a few large papules. Itching severe at night. Under liquid pitch and diachylon ointment this trouble was rapidly relieved. About this time there developed the typical lesions of ringworm, and thenceforth these were appropriately treated. His brother was similarly affected.

RINGWORM OF THE SCALP.

The following case of a boy, aged 6 years, is characteristic. Present about a month. On the right parietal eminence a patch nearly the size of the palm, roundish, reddened, slightly scaly, nearly bald, only a few broken, loose hairs present, save near borders. Had been treated by some unknown applications. The fungus was demonstrated in the hairs. This boy was well in about six weeks.

RINGWORM OF THE BEARD.

Farmer, aged 26. Disease on hairy parts of cheeks, upper lip and chin. Began on the upper lip nearly a year and a half before. There were, when examined, elevated crusts enclosing clumps of hair, and some more diffuse, flat, yellowish-brown crusts, with here and there the brawny inflammatory condition which is quite characteristic. Between the crusts a more or less grayish, scaly condition of the skin. Cicatricial baldness on right cheek. No visible change in hairs of the two weeks' old beard. Condition was probably aggravated by previous strong applications. I believed that I demonstrated the fungus in the root-sheaths of hairs extracted. Owing to patient's disinclination to keep cleanly shaven and thus allow the applications to reach the skin, and to the inherent obstinacy of the disease, very little was accomplished in the treatment of this case.

TREATMENT.

I have reserved all mention of treatment to the last.

The first case which I treated in Atlanta was a case of ringworm of the scroto-femoral region. For the preceding two years I had had more to do with carrying out other people's prescriptions than with writing them. So I forgot whether it was *chrysorobin* or *pyrogallic acid*, the latter of which I have never seen prescribed for it, that was "good" in ringworm. I

ordered the wrong one in theory, but that one prescription convinced me there was nothing better for cutaneous ringworm. I wrote the following :

R. Acid pyrogallic, gr. xv.
Collodii, ℥i.

M. Sig.—Paint on often enough to keep lesions covered.

The few cases of ordinary ringworm of the skin and the larger number of scroto-femoral ringworm have all yielded quite promptly to this treatment. It may require to be weakened or increased in strength, according to the quality of skin upon which it is used. Whether the irritative action of pyrogallic acid does the work or the exclusion of air by the collodion, or whether the former destroys the fungus, I do not know.

For ringworm of the scalp I have used bichloride of mercury, one to two grains, kerosene oil one ounce. This combination was mentioned to me by Dr. Elliot, of New York, last spring, and he reported excellent results from its use in institution practice. In October, 1891, Dr. C. G. Kerly reported (New York *Medical Journal*) 31 cases successfully treated with the following : Kerosene and olive oil, aa. 15; bichloride, one-eighth of 1 part; alcohol, sufficient to solution. Irritation produced was treated with simple salves. (The gentleman reporting this article for Unna's long-named, German Skin *Journal*, says it was not mentioned whether these were old or new cases, and that it was well known that irritants can cure a recent case.) The bichloride in kerosene alone is doubtfully soluble, but the effect has been perfectly satisfactory. The irritation produced was only slight and accompanied by the formation of thin plates of scale covering the parts treated, but in one case, predisposed to eczema, there was a diffuse eczematous outbreak upon the scalp. In none of the cases was the commonly recommended epilation practiced.

Various treatment was used on the single case of ringworm of the beard, but the results were not such as to justify detailing the treatment here.

